# Unexpected binding behaviors of bacterial Argonautes in human cells cast doubts on their use as targetable gene regulators

**DOI:** 10.1371/journal.pone.0193818

**Published:** 2018-03-27

**Authors:** Henriette O’Geen, Chonghua Ren, Nicole B. Coggins, Sofie L. Bates, David J. Segal

**Affiliations:** 1 Genome Center, University of California, Davis, California, United States of America; 2 Department of Biochemistry and Molecular Medicine, University of California, Davis, California, United States of America; Hirosaki University Graduate School of Medicine, JAPAN

## Abstract

Prokaryotic Argonaute proteins (pAgos) have been proposed as an alternative to the CRISPR/Cas9 platform for gene editing. Although Argonaute from *Natronobacterium gregoryi* (*Ng*Ago) was recently shown unable to cleave genomic DNA in mammalian cells, the utility of *Ng*Ago or other pAgos as a targetable DNA-binding platform for epigenetic editing has not been explored. In this report, we evaluated the utility of two prokaryotic Argonautes (*Ng*Ago and *Tt*Ago) as DNA-guided DNA-binding proteins. *Ng*Ago showed no meaningful binding to chromosomal targets, while *Tt*Ago displayed seemingly non-specific binding to chromosomal DNA even in the absence of guide DNA. The observed lack of DNA-guided targeting and unexpected guide-independent genome sampling under the conditions in this study provide evidence that these pAgos might be suitable for neither gene nor epigenome editing in mammalian cells.

## Introduction

Eukaryotic Argonaute proteins (eAgos) use small single-stranded RNA to target complimentary RNA sequences and play a key role in the RNA interference (RNAi) pathway [1]. On the other hand, recently discovered prokaryotic Argonaute proteins (pAgos) have been implicated in the targeting of foreign DNA for degradation [2]. Some pAgos have been shown to use small single-stranded DNA (ssDNA) to target and cleave double-stranded DNA and hence offer an intriguing possibility for gene and epigenome editing [3–6].

**Fig 1:**
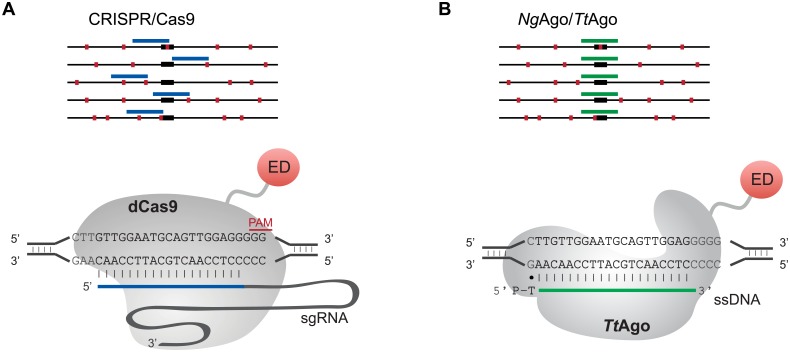
The potential for high-precision targeting by pAgos. A. Targeting of CRISPR/Cas9 is limited by the requirement of a PAM site (green boxes). B. *Ng*Ago or *Tt*Ago might allow the precise targeting of features, such as transcription factor binding sites (black boxes). gRNA/gDNA, red lines.

In principle, pAgos offer several advantages for DNA-guided site-specific binding in the mammalian genome, particularly because of their increased flexibility of targeting. While CRISPR/Cas9 is able to target near many genetic features, the necessity of a protospacer adjacent motif (PAM) in the target site often makes it impossible to design guide RNAs (gRNAs) to bind exactly at features such as SNPs, individual CpGs, intron/exon boundaries, or specific transcription factor binding sites (Fig 1A). Indeed this limitation has led to many efforts to expand the targeting lexicon using engineered or natural variant Cas9 proteins [7–10]. However, nearly all have been similarly or even more restricted than the 5’-NGG-3’ PAM site of the canonical *Sp*Cas9 [11]. pAgos do not require a PAM adjacent to their guide DNA (gDNA) target sites, raising the prospect of targeting without sequence restrictions (Fig 1B).

An early report that the Argonaute from *Natronobacterium gregoryi* (*Ng*Ago) could efficiently cleave genomic DNA in mammalian cells [12] was found to be irreproducible [13–17], eventually leading to its retraction [18]. The latter studies suggest that *Ng*Ago will not be a useful tool for gene editing. However they did not rule out that *Ng*Ago and other pAgos could be used as targetable DNA binding platforms, in analogy to catalytically inactive or dead Cas9 (dCas9) [19–21] or Cpf1 (dCpf1) [22, 23]. dCas9 has been widely used for applications such as gene activation or repression, epigenome editing, and imaging of DNA and RNA in living cells (reviewed in [24, 25]). Indeed, two recent studies found that *Ng*Ago was able to inhibit gene expression even in the absence of DNA editing [15, 17], reminiscent of dCas9-mediated CRISPR interference (CRISPRi, [19]).

These observations motivated us to examine *Ng*Ago as a PAM-less, DNA-guided DNA binding platform. In addition, pAgo from *Thermus thermophilus* (*Tt*Ago) can be programmed to bind and cleave double stranded plasmid in a site-specific manner *in vitro* [4, 5]. The use of *Tt*Ago as a tool for gene editing in mammalian cells is limited by its requirement for high temperatures to cleave double-stranded DNA. However, it has been shown to bind and cut single-stranded DNA at a physiological temperature of 37°C [5], making *Tt*Ago an additional candidate as a programmable DNA-binding platform.

## Results

### Confirmation of lack of targeted DNA cleavage by h*Tt*Ago and h*Ng*Ago in the mammalian genome

**Fig 2:**
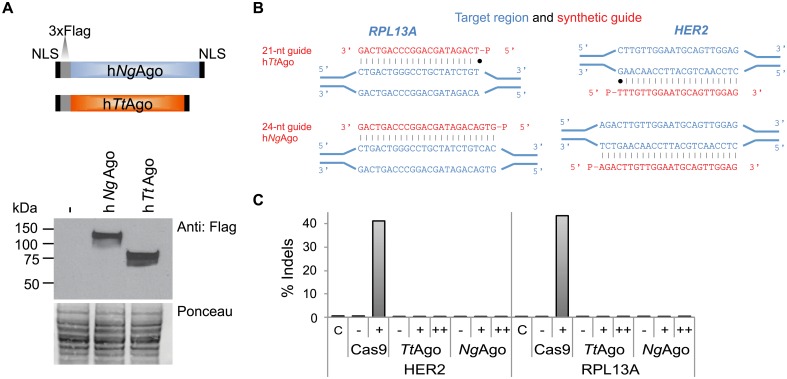
h*Tt*Ago and h*Ng*Ago do not cleave genomic target sites. A. Schematic of human codon-optimized *Tt*Ago and *Ng*Ago proteins containing two nuclear localization signals (NLSs) and a 3xFlag epitope tag. Western blot analysis of h*Tt*Ago and h*Ng*Ago proteins in HEK293 cells using an antibody against the 3xFlag tag. Untransfected cells serve as a negative control (-). Ponceau staining was used as a loading control. B. Diagram illustrating *RPL13A* and *HER2* guide DNAs and genomic target site. Genomic target sites are indicated in blue and complementary ssDNA guides are indicated in red. C. Amplicon sequencing was carried out on HEK293 cells co-transfected with h*Tt*Ago or h*Ng*Ago expression plasmids with (+) or without (-) gDNAs to *RPL13A* and *HER2*. To increase the amount of gDNAs, cells were re-transfected with gDNAs 24 hours after the initial transfection (++). CRISPRESSO analysis confirms that h*Tt*Ago and h*Ng*Ago did not cause insertions or deletions (indels) under any of these conditions (S5 Table). As a control, HEK293 cells were co-transfected with Cas9 nuclease and gRNA expression plasmids targeting *RPL13A* and *HER2*. RNA-guided Cas9 displayed target site cleavage at the genomic *RPL13A* and *HER2* target sites. The percentage of sequence reads containing indels relative to the total number of sequence reads is plotted on the y-axis.

First we evaluated that *Tt*Ago and *Ng*Ago expressed in mammalian cells are in principle able to cleave DNA targets *in vitro*. HEK293 cells were transfected with plasmids expressing human codon optimized Argonautes (h*Ng*Ago and h*Tt*Ago) with an N-terminal 3xFlag epitope tag and nuclear localization signals both at the N and C termini (Fig 2A; protein sequence provided as S1 Fig). Western blot analysis confirmed h*Tt*Ago and h*Ng*Ago protein expression in HEK293 cells (Fig 2B). h*Tt*Ago and h*Ng*Ago proteins were purified from HEK293 cells (S2A Fig) and the nature of co-purified nucleic acids was evaluated (S2B Fig). The profile of nucleic acids co-purified with mammalian expressed h*Tt*Ago recapitulates *Tt*Ago binding characteristics observed in *E*. *coli* [5]. The majority of co-purified nucleic acids consisted of RNA, but in addition short DNAs were also observed. The ability of purified h*Tt*Ago/h*Ng*Ago to cleave single-stranded DNA templates was assessed *in vitro* (S2C Fig, S1 Table). As expected, h*Tt*Ago cleaved a 98-nt single-stranded DNA target when provided with a 21-nt phosphorylated gDNA (previously described in [5]) at the high temperatures of 75°C and 55°C (S2D and S2E Fig). However, no cleavage activity was observed at the physiological temperature of 37°C (S2F Fig). It has recently been shown that bacterial expressed *Tt*Ago has a preference for target sites with a G nucleotide in position 1 (t1G; [26]). Cleavage efficiency of mammalian-expressed h*Tt*Ago at the *RPL13A* target site was only detectable when the target site had a G nucleotide in position 1 (t1G; S2E Fig). No DNA cleavage was observed for h*Ng*Ago (S2D–S2F Fig).

As it is a prerequisite for transcriptional and epigenetic regulators to bind but not cleave their target sequence *in vivo*, we evaluated the cleavage ability of h*Tt*Ago in HEK293 and HeLa cells at six genomic target sites (*NFE2L1*, *NPAS1*, *RPL13A* site1, *RPL13A* site2, *RB1A* site1, *RB1A* site2) using three gDNA configurations: 1) a single gDNA, 2) two complimentary gDNAs and 3) two gDNAs targeting the opposite DNA strand separated by a 15-nt spacer (S3 Fig, gDNAs are listed in S2 Table). An h*Tt*Ago-expressing plasmid was transfected alone or in combination with different 5’-phosphorylated 21-nt gDNAs in HEK293 cells. Amplicon sequencing was used to capture even low frequency cleavage events. Genomic target regions were amplified by PCR (primer sequences are listed in S3 Table) and analyzed for the presence of insertions and deletions (indels) at the cleavage site using next-gen sequencing. We did not observe indel frequencies above background, or above the detection limit of 0.01%, at any of the regions targeted by h*Tt*Ago (S4 Table). After adjusting for read depth, the number of substitutions were similar between untreated cells, cells that expressed h*Tt*Ago only and cells that contained both h*Tt*Ago and gDNAs. The same results were obtained when the 5’-phosphorylated gDNAs contained four phosphorothioate linkages to prevent degradation of the gDNAs (S5 Table).

Several studies have reported that *Ng*Ago is not able to cleave genomic DNA *in vivo* [13–17]. When we expanded indel analysis to include h*Ng*Ago at the *HER2* and *RPL13A* loci in HEK293 cells, we did not observe cleavage by h*Tt*Ago or h*Ng*Ago in the presence of 21-nt or 24-nt gDNAs, respectively (S6 Table). To increase the amount of gDNAs present in the cell, we re-transfected cells with their respective DNA guides 24 hours after the initial co-transfection with pAgo and gDNAs. Increase of gDNAs in the cell did not result in DNA cleavage at the corresponding genomic target (Fig 2C). When *HER2* and *RPL13A* sites were targeted with *Sp*Cas9-gRNA complexes, cleavage efficiencies of 40% and 43% were observed, respectively (Fig 2C).

### 5’ Phosphorylated single-strand DNA guides fail to guide h*Ng*Ago and h*Tt*Ago to specific genomic target sites

**Fig 3:**
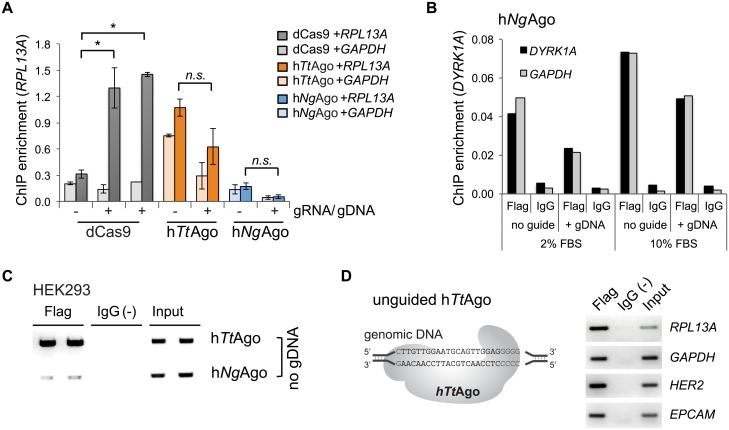
DNA guides do not facilitate binding of h*Tt*Ago and h*Ng*Ago to genomic target loci. A. ChIP-qPCR enrichment to quantitate binding to *RPL13A* locus. HEK293 cells were transfected with h*Tt*Ago- and h*Ng*Ago-expression plasmids either with (+) or without (-) gDNAs. dCas9 and two different gRNA expressing plasmids were co-transfected as controls (Student two-sided *T*-test; *, *p*<0.05; *n*.*s*., not significant; *n* = 2 independent experiments; mean ± SEM). Binding to a non-target locus (GAPDH) was evaluated to interrogate binding specificity. B. Targeted binding of h*Ng*Ago failed at the *DYRK1A* locus in HEK293 cells. HEK293 cells were co-transfected with h*Ng*Ago expression plasmid together with 24-nt 5’-phosphorylated gDNAs (G5 gDNA) or without gDNA. ChIP assays were performed with an antibody to the 3xFlag tag of h*Ng*Ago or with rabbit IgG as a negative control. Addition of G5 gDNA that was targeted to the *DYRK1A* locus did not increase h*Ng*Ago binding above background level (no guide). There was no difference in binding to *DYRK1A* or to a control region (*GAPDH*). Two conditions (2% or 10% FBS in growth media) were tested. C. ChIP-PCR to measure unguided h*Tt*Ago and h*Ng*Ago binding to the *RPL13A* locus. ChIP assays were performed with two biological replicates. Flag, Flag ChIP; IgG(-), IgG negative control; Input, 0.1% chromatin input. D. h*Tt*Ago binds multiple genomic regions without gDNA in HeLa cells. ChIP assays were performed in HeLa cells expressing h*Tt*Ago without DNA guides. Standard PCR with locus-specific primers demonstrated h*Tt*Ago binding to *RPL13A*, *GAPDH*, *HER2* and *EPCAM*. Flag, Flag ChIP; IgG(-), IgG negative control; Input, 0.1% chromatin input.

Having established that the pAgos did not cleave their genomic target sites, we next investigated if gDNAs can guide h*Tt*Ago or h*Ng*Ago to complementary genomic target sites in mammalian cells. We performed chromatin immunoprecipitation (ChIP) followed by quantitative PCR (ChIP-qPCR) using an antibody to the 3xFlag tag of h*Ng*Ago or h*Tt*Ago in the presence or absence of 5’ phosphorylated gDNA to the *RPL13A* locus in HEK293 cells. As a positive control, we used a catalytically inactive Cas9 (dead or dCas9) and a gRNA targeting *RPL13A*. As expected, dCas9 was able to bind the target locus in a gRNA-dependent manner, resulting in a 4-fold increase of dCas9 binding to the sense or anti-sense strand of the *RPL13A* locus in the presence of the gRNA (Fig 3A, Student’s t-test p<0.05). Binding of dCas9 was specific, since *RPL13A* gRNA did not increase dCas9 binding at the *GAPDH* control locus. However, the binding of h*Ng*Ago to *RPL13A* was similar to the background level of Cas9 binding, and there was no preference for the target site compared to the non-target *GAPDH* control locus (Fig 3A). Similar results were observed when h*Ng*Ago was targeted to *DYRK1A* using the G5 gDNA described in the original report by Gao *et al*. [12], in the presence of either 2% or 10% FBS-supplemented growth media (Fig 3B). ChIP enrichment for h*Ng*Ago was always greater than for the IgG negative control, indicating some binding to chromosomal DNA (Fig 3B and 3C). However, the binding was weak, non-specific, and occurred even in the absence of a gDNA. In fact, the presence of a gDNA appeared to cause a slight decrease in DNA binding, although the trend did not reach the level of statistical significance. In contrast, h*Tt*Ago displayed strongly enriched binding to chromosomal DNA in HEK293 cells (Fig 3A and 3C). However, there was no significant preference for the *RPL13A* target compared to the non-target *GAPDH*, and binding occurred in the absence of a gDNA (Fig 3A). The unexpected affinity of h*Tt*Ago for untargeted genomic sites was further evaluated by ChIP-qPCR in HeLa cells at the *RPL13A*, *EPCAM*, *HER2* and *GAPDH* loci. Strikingly, in the absence of any transfected gDNA, binding of h*Tt*Ago was observed at all loci investigated (Fig 3D).

## Discussion

In this study, we evaluated the utility of pAgos as DNA-guided DNA-targeting tools using ChIP assays. Neither h*Ng*Ago nor h*Tt*Ago were able to bind to the mammalian genome in a targeted manner, but instead appeared to bind to essentially all loci examined in two human cell types, even in the absence of any gDNA. Overall our data demonstrate that single-stranded gDNAs do not efficiently guide h*Tt*Ago or h*Ng*Ago to specific genomic sites in the physiological context of a mammalian cell. We have demonstrated that the mammalian-expressed h*Tt*Ago is functional in an *in vitro* cleavage assay. This is important to note since nuclear localization signals have been appended to the N- and C-terminal ends of h*Tt*Ago. The C-terminal amino acid of h*Tt*Ago and the Argonaute from *Archaeoglobus fulgidus* is structurally important for the binding of magnesium and the DNA or RNA guide [27, 28]. We were unable to observe evidence of cleavage using mammalian-expressed h*Ng*Ago either *in vitro* or *in vivo*. By now, several groups have reported that *Ng*Ago cannot cleave genomic DNA *in vivo* [13–17], but *in vitro* cleavage assays have not been reported. Our results lead us to conclude that h*Ng*Ago and h*Tt*Ago may not be suitable as targetable DNA-binding platforms for creating epigenetic editing tools. It is possible that some aspect of the experimental conditions prevented more favorable outcomes, such as unexpectedly poor transfection of guide DNAs. However, we note that the conditions used here did support binding of d*Sp*Cas9, and were the same used by others to show that *Ng*Ago could not cleave DNA *in vivo*. Exploration of mutations or other variants of Argonaute proteins or covalently linking gDNAs to Argonaute proteins may provide a more positive outcome.

The mechanism for the dramatic unguided and seemingly non-specific binding is unclear. One explanation could be the sampling and cleavage of foreign DNA by pAgos that has been proposed as a means of host defense against mobile DNA from viruses or plasmids [5, 26, 29, 30]. The mechanism by which pAgos acquire guides to preferentially target foreign DNA remains elusive, but presumably could be operating in the mammalian cells to cause apparent non-specific binding to DNA sequences. The *Tt*Ago double mutant (D478A/D546A) that is unable to bind small single-stranded DNA guides may be useful for testing such a hypothesis [5]. Another explanation could be that some pAgos simply have non-specific DNA binding activity (*i*.*e*., are sticky). In this regard, it is worth noting that h*Tt*Ago, which showed the greatest binding activity, comes from a thermophilic species and may therefore have surface charges or other features that assist in its binding to DNA at high temperatures. These features might result in non-specific binding at lower temperatures. Alternatively, the pAgos might interact with unknown proteins or RNA in human cells.

Our findings would also seem to rule out a CRISPRi-like mechanism to explain the observed knock-down by *Ng*Ago of *fabp11a* mRNA expression in zebrafish embryos [15] and hepatitis B virus pgRNA in human cells [17]. It is possible that *Ng*Ago induced DNA-guided gene knockdown in these cases through a DNA-dependent RNA cleavage activity [31]. Prokaryotic Argonautes have been the subject of exuberant expectations and dramatic disappointments regarding their use as targeted tools to alter gene expression. However, continued careful study and characterization may yet provide the mechanistic insights required for their appropriate application.

## Materials and methods

### Construction of h*Tt*Ago and h*Ng*Ago expression plasmids

The human codon-optimized *Tt*Ago was synthesized from GeneArt. The GeneArt plasmid was digested with *Sfi*I restriction enzyme. pCDNA3-hTtAgo was created using Gibson Assembly (New England Biolabs) by inserting the h*Tt*Ago fragment into the *Fse*I and *Nhe*I digested ideal pCDNA3-dCas9 expression plasmid replacing the dCas9 sequence [32]. The human codon-optimized *Ng*Ago was synthesized in two gBlocks (IDT) and was cloned into *Kpn*I and *Nhe*I digested pCDNA3-hTtAgo by Gibson Assembly of the three fragments (New England Biolabs). The resulting pCDNA3-hTtAgo and pCDNA3-hNgAgo expression plasmids contain two nuclear localization signals (NLS) and a 3x Flag epitope tag (S1 Fig). Plasmids and annotated GenBank files are available through Addgene.

### Design of gDNAs and gRNA expression vector

gDNAs and gRNAs were designed to the same genomic sequence. Single-stranded guide DNA sequences were 21 or 24 nucleotides in length for h*Tt*Ago and h*Ng*Ago, respectively and carried 5’-phosphorylation or an additional four phosphorothioate linkages (S1 and S2 Tables). Plasmids for gRNA expression were cloned as previously described [33]. Briefly, the empty gRNA cloning vector (Addgene plasmid # 41824) was linearized using the *Afl*II restriction enzyme. 19-bp gRNA target sequences (19N) were selected and the G-19N sequence incorporated into two 60mer oligonucleotides that contained cloning vector overhangs for Gibson assembly. After annealing and extending the oligonucleotides to 100-bp, the PCR reaction was purified (PCR purification kit; QIAGEN) and dsDNA was Gibson assembled into the *Afl*II linearized plasmid. Oligonucleotide sequences of gRNA target sites are listed in S2 Table.

### Cell lines and transfection

The human cell lines HEK293-c18 (ATCC #CRL-10852) and HeLa (ATCC #CCL-2) were grown in Dulbecco’s modified Eagle’s medium (DMEM) supplemented with 10% fetal bovine serum (FBS) and 1% penicillin/streptomycin. Cells were maintained at 37°C and 5% CO_2_. Cells were transfected at ~70% confluency using Lipofectamine 3000 (Life Technologies) following the manufacturer’s instructions. For indel analysis, transfections were performed in 24-well dishes using 250 ng h*Tt*Ago or h*Ng*Ago expression plasmid and 1 μl of 100 μM gDNA per well. Alternatively, cells were co-transfected with 250 ng hCas9 nuclease (Addgene plasmid #41815) and 250 ng gRNA expressing plasmids. Cells were harvested 72 hours after transfection. For ChIP assays, transfections were carried out in 10-cm dishes using 2.5 μg pCDNA3-h*Tt*Ago or pCDNA3-h*Ng*Ago with or without 7.5 μl of 100 μM gDNA. Samples that were not co-transfected with gDNA, were instead co-transfected with 7.5 μg of plasmid pBABE-puro (Addgene plasmid #1764). Alternatively, cells were co-transfected with 2.5 μg pCDNA3-dCas9 and 7.5 μg of gRNA expressing plasmid.

### *Ng*Ago and *Tt*Ago expression and purification from mammalian cells

HEK293-c18 cells were grown in 10 cm dishes and transfected with 10 μg h*Tt*Ago or h*Ng*Ago expression plasmid using Lipofectamine 3000 (Life Technologies) following the manufacturer’s instructions. 60 hours post transfection cells were rinsed once with PBS and protein was extracted using 1 mL 1xRIPA buffer (Millipore) supplemented with protease inhibitor cocktail (Roche). Anti-Flag M2 affinity gel (Sigma) was equilibrated by washing three times with 1mL TBS (50 mM Tris-HCl pH 7.5, 150 mM NaCl). 50 μl of equilibrated anti-Flag affinity gel was added to 1 mL RIPA lysate and incubated on a rotating platform for 2 hours at 4°C. Anti-Flag affinity gel was washed three times with 1mL TBS and bound protein was eluted with either 3XFlag peptide or 0.1 M glycine. Flag tagged protein was eluted in 100 μl TBS with 150 ng/μl 3X Flag peptide (SIGMA) by rocking at 4°C for 30 minutes and was then used for Western blot analysis and *in vitro* activity assay. For co-purification of nucleic acids, protein was eluted using 0.1 M glycine.

### Extraction of nucleic acids co-purified with h*Tt*Ago

100 μl purified h*Tt*Ago obtained from one 10-cm dish of transfected HEK293 cells were incubated with 1 μl proteinase K for 1 hour at 37°C and separated into three reactions. Two samples were treated with RNase A or DNase I, respectively for 30 minutes at 37°C. The third sample was kept untreated. Nucleic acids were extracted using phenol/chloroform with an additional step of chloroform back extraction to increase recovery. Nucleic acids were precipitated (0.75M NH4OAc, 70% ethanol) and washed twice with 80% ethanol. The dried pellet was dissolved in TBE urea sample buffer (ThermoFisher) and heated for 5 minutes at 95°C. Nucleic acids were separated on a 15% Novex TBE-Urea Gel (ThermoFisher) and visualized with ethidium bromide staining.

### Activity assay

15 μl purified h*Tt*Ago or h*Ng*Ago were mixed with 5 pmoles single-stranded DNA guide and 5 pmoles 98-nt ssDNA target (S6 Table) in assay buffer (20 mM Tris-HCl pH8, 250 mM NaCl supplemented with 5 mM MnCl_2_) and incubated for one hour at 75°C, 55°C or 37°C. The reaction mixture was treated with 10 μg RNase A. The reaction was stopped by addition of TBE urea sample buffer (ThermoFisher) and heated for 5 minutes at 95°C. Samples were resolved on a 15% Novex TBE-Urea Gel (ThermoFisher) and ssDNA was visualized by staining with ethidium bromide.

### Amplicon sequencing

72 hours post-transfection with pCDNA3-h*Tt*Ago and phosphorylated 21-nt gDNAs, genomic DNA was extracted using Quick-DNA Miniprep Kit (Zymo Research). Non-transfected cells and cells only transfected with pCDNA3-h*Tt*Ago were used as a control. 100ng genomic DNA was used for PCR amplification with GoTaq polymerase (Promega). For the experiment with h*Tt*Ago, oligonucleotide sequences amplifying 270-bp to 285-bp target regions are listed in S3 Table. PCR products were purified using the QIAquick PCR Purification (Qiagen) and six unique amplicons were mixed in equal amounts per amplicon pool. Illumina sequencing libraries were generated from individual amplicon pools (from different treatments or different cell types) by ligating barcoded adapters (Bioo Scientific) using T4 ligase (New England Biolabs) following the manufacturer’s instructions. Sequencing libraries were cleaned up using 0.8x Ampure XP beads (Agencourt) and library concentrations were determined with the Qubit^™^ dsDNA HS Assay kit (Invitrogen). Equal amounts of amplicon pools were mixed and sequenced in one lane of paired end 250 on the MiSeq (Illumina). Sequencing and demultiplexing of amplicons pools was performed at the DNA Technologies and Expression Analysis Cores at the UC Davis Genome Center. Results are summarized in S4 and S5 Tables. To compare cleavage efficiency of DNA-guided h*Tt*Ago and h*Ng*Ago (21-nt and 24-nt gDNAs, respectively) to that of RNA-guided *Sp*Cas9, genomic DNA was extracted 72 hours post-transfection using Quick-DNA Miniprep Kit (Zymo Research). 100ng genomic DNA was used for PCR amplification of ~200-bp fragments with Taq RED DNA Polymerase Master Mix (Apex) following the manufacturer’s recommendations. Forward primers contain a 5-nt barcode for multiplexing amplicons (S3 Table). All amplicons were purified using QIAQuick PCR Purification Kit (Qiagen) and pooled at equal concentrations for Illumina sequencing. Sequencing library preparation and amplicon sequencing were performed by the CCIB DNA Core Facility at Massachusetts General Hospital (Cambridge, MA). Results are summarized in S6 Table.

### Amplicon sequencing data analysis

Sequencing data was processed using FLASH2 (https://github.com/dstreett/FLASH2) to overlap forward and reverse reads and merge paired reads into a single long read. For amplicons containing a 5-nt barcode, we demultiplexed merged reads with the FASTX barcode splitter by identifying barcodes at the beginning or the reverse complement barcodes at the end of sequence reads, allowing for one mismatch. Processed fastq files were analyzed with the CRISPResso online tool (http://crispresso.rocks) using default settings.

### Chromatin immunoprecipitation (ChIP)

For ChIP assays transfected cells were cross-linked 48 hours post transfection by incubation with 1% formaldehyde solution for 10 min at room temperature and the reaction was stopped by the addition of glycine to a final concentration of 125 mM. Cross-linked cell pellets were stored at -80°C. Chromatin was extracted and ChIP performed using StaphA cells (Sigma-Aldrich, St. Louis, MO, USA) to collect the immunoprecipitates as previously described [33]. In summary, cells were lysed in Cell Lysis Buffer (5mM PIPES, 85mM KCl, 1% Igepal, 1x Protease Inhibitors; pH 8.0) on ice. Nuclei were pelleted and lysed for 30 minutes in Nuclear Lysis Buffer (50mM Tris-HCl, 10mM EDTA, 1% SDS, 1x Protease Inhibitors; pH 8.0) on ice. Chromatin was sheared to an average fragment size of 500-bp using a Bioruptor 2000 (Diagenode). Chromatin from an entire 10-cm dish was split into Flag and control ChIP assay and diluted with 3 volumes of IP Dilution Buffer (16.7mM Tris-HCl, 167mM NaCl, 1.2mM EDTA pH 8.0, 1.1% Triton X 100, 0.01% SDS, 1x Protease Inhibitors). ChIP enrichment was performed by incubation with 3 μg anti-Flag antibody (SIGMA M2 F1804) or 2 μg normal rabbit IgG (Abcam ab46540) for 16 h at 4 °C. Immuno complexes were captured with 3 μg rabbit anti mouse antibody for 1 hour at 4°C and were bound to StaphA cells for 15 minutes at room temperature. Chromatin-antibody complexes were pelleted and washed twice with Wash Buffer 1 (50mM Tris-HCl, 2mM EDTA pH 8.0, 0.2% Sarkosyl) and four times with Wash Buffer 2 (100mM Tri-HCl, 500mM LiCl, 1% Igepal, 1% Deoxycholic Acid; pH 8.0). Immunoprecipitated chromatin was eluted with 100 μl ChIP Elution Buffer (50mM NaHCO3, 1% SDS) by shaking at room temperature for 15 minutes. Crosslinks were reversed at 67°C overnight after addition of 12 μl 5M NaCl. Finally, immunoprecipitated DNA was treated with 10 μg RNase A at 37°C for 20 minutes, and purified using the QIAQuick PCR Purification Kit (Qiagen). ChIP-DNA and diluted Input control were used for subsequent qPCR or standard PCR reactions (Primers are listed in S3 Table). qPCR was carried out with 2x SYBR FAST mastermix (KAPA Biosystems) according to the manufacturer’s recommendations using the CFX384 Real-Time System C1000 Touch Thermo Cycler (BioRad). ChIP enrichment was calculated relative to input samples using the dCq method (dCq = Cq[ChIP]-Cq[input]). We applied the Student’s paired t-test with a two-tailed distribution to determine statistical significance for ChIP enrichment without or with gDNA/gRNA. Standard PCR was performed using GoTaq (Promega) DNA polymerase (2 min at 95°C; 30 sec at 95°C, 30 s at 55°C, 30 s at 72°C, 35 cycles; 5 min at 72°C). PCR products were separated on a 1.5% agarose gel and visualized using the Gel Doc^™^ XR+ System (BioRad).

### Western blot analysis

HEK293-c18 cells were transfected in 6-well dishes with 2.5 μg pCDNA3-h*Tt*Ago or pCDNA3-h*Ng*Ago and lysed 48 hours post transfection in 1xRIPA buffer (Millipore) supplemented with protease inhibitor cocktail (Roche). Protein concentrations were determined by Bradford assay (BioRad) and 20 μg protein was separated on a 10–20% NuPAGE Bis-Tris gel (Thermo Fisher Scientific) in MOPS buffer and transferred onto nitrocellulose membranes. Protein loading was evaluated by Ponceau S stain. After rinsing the membrane with deionized water, non-specific antigen binding was blocked in TBST (50 mM Tris, 150 mM NaCl and 0.1% Tween-20) with 5% nonfat dry milk (Cell Signaling). Membranes were incubated with monoclonal antibody against Flag (1:1500; SIGMA M2 F1804) in blocking solution at 4°C over night. Membranes were washed with TBST three times for 10 minutes before incubation with anti-mouse HRP-conjugated antibody at room temperature. After 45 minutes, the membrane was washed three times in TBST and proteins were visualized with Amersham ECL Prime Western Blotting Detection Reagent (GE Healthcare) and autoradiobiography film.

## Supporting information

S1 Fig Protein sequences of h*Ng*Ago and h*Tt*Ago used in this study. (PDF)

S2 Fig h*Tt*Ago cleaves ssDNA using ssDNA guides *in vitro*. A. h*Tt*Ago was expressed in HEK293 cells and purified by immunoprecipitation (IP) with Flag antibody. A control IP was performed using rabbit IgG. Protein extract from transfected cells was loaded as a control. B. Copurification of nucleic acids with hTtAgo in HEK293 cells. Nucleic acids were resolved on a 15% TBE-urea gel. Nucleic acids were not treated (lane 1) or treated with RNaseA (lane 2) or DNaseI (lane 3). C. 21-nucleotide (nt) DNA guides are complimentary to 98-nt single stranded DNA target. Predicted cleavage sites are indicated by a black triangle. D-F. Purified h*Tt*Ago or h*Ng*Ago were incubated with 21-nt or 24-nt guides, respectively to cleave a 98-nt ssDNA target and run on a 15% TBE-urea gel. Cleavage assays were carried out at 75°C (A), 55°C (B) and 37°C (C). Control Flag-IPs were performed in untransfected HEK293 cells. h*Tt*Ago was incubated with the a DNA guide complimentary to a G nucleotide in position 1 on the target strand (t1G) as indicated.
(PDF)

S3 Fig Design of h*Tt*Ago guide DNAs. Diagram illustrating the three different gDNA constellations that were tested: forward (FW) guide only, forward (FW) and reverse (RV) guides or forward (FW) and reverse (RV) guides separated by a 15-nt spacer sequence. The genomic RPL13A target site is indicated in blue and complementary ssDNA guides are indicated in red.
(PDF)

S1 Table List of oligonucleotide sequences for activity assay. (PDF)

S2 Table List of oligonucleotide sequences of guide DNAs and gRNA target sites. (PDF)

S3 Table List of oligonucleotide sequences for amplification of genomic target regions. (PDF)

S4 Table Indel analysis of PCR amplified genomic target sites using 5'-phosphorylated guide DNAs. (PDF)

S5 Table Indel analysis of amplified genomic targets using 5'-phosphorylated gDNAs with phosphorothioate modifications. (PDF)

S6 Table Indel analysis of amplified genomic target sites comparing cleavage ability of RNA-guided SpCas9 and DNA-guided *Tt*Ago and *Ng*Ago in HeK293-c18 cells. (PDF)
