# Supplementary material for: Unexpected binding behaviors of bacterial Argonautes in human cells cast doubts on their use as targetable gene regulators
Source: PLoS One. 2018 Mar 27;13(3):e0193818. doi: 10.1371/journal.pone.0193818 (PMC5870970; doi:10.1371/journal.pone.0193818)
Supplement: S1 Fig — (PDF) [file pone.0193818.s001.pdf]

h*Ng*Ago

NLS (SV40): aa 2-9
3X Flag: aa 15-36
hNgAgo: aa 65-764
NLS (Neoplasmin): aa 977-992
P2A cleavage peptide: aa 999-1017

MPKKKRKVGGSGGSDYKDHDGDYKDHDIDYKDDDDKGGGSGGGSGTGGSGGSGGSGGSGGSGRPMTVIDLDSTTTADE
LTSGHTYDISVTLTGVYDNTDEQHPRMSLAFEQDNGERRYITLWKNTTPKDVFTYDYATGSTYIFTNIDYEVKDGYEN
LTATYQTTVENATAQEVGTTDEDETFAGGEPLDHHLDDALNETPDDAETESDSGHVMTSFASRDQLPEWTLHTYTLTA
TDGAKTDTEYARRTLAYTVRQELYTDHDAAPVATDGLMLLTPEPLGETPLDLDCGVRVEADETRTLDYTTAKDRLLAR
ELVEEGLKRSLWDDYLVRGIDEVLSKEPVLTCDEFDLHERYDLSVEVGHSGRAYLHINFRHRFVPKLTLADIDDDNIY
PGLRVKTTYRPRRGHIVWGLRDECATDSLNTLGNQSVVAYHRNNQTPINTDLLDAIEAADRRVVETRRQGHGDDAVSF
PQELLAVEPNTHQIKQFASDGFHQQARSKTRLSASRCSEKAQAFAERLDPVRLNGSTVEFSSEFFTGNNEQQLRLLYE
NGESVLTFRDGARGAHPDETFSKGIVNPPESFEVAVVLPEQQADTCKAQWDTMADLLNQAGAPPTRSETVQYDAFSSP
ESISLNVAGAIDPSEVDAAFVVLPPDQEGFADLASPTETYDELKKALANMGIYSQMAYFDRFRDAKIFYTRNVALGLL
AAAGGVAFTTEHAMPGDADMFIGIDVSRSYPEDGASGQINIAATATAVYKDGTILGHSSTRPQLGEKLQSTDVRDIMK
NAILGYQQVTGESPTHIVIHRDGFMNEDLDPATEFLNEQGVEYDIVEIRKQPQTRLLAVSDVQYDTPVKSIAAINQNE
PRATVATFGAPEYLATRDGGGLPRPIQIERVAGETDIETLTRQVYLLSQSHIQVHNSTARLPITTAYADQASTHATKG
YLVQTGAFESNVGFLGGSGGSGGSGGSGGSASGGGSGGGSKRPAATKKAGQAKKKKGGSGSGATNFSLLKQAGDVEEN
PGPAAA

h*Tt*Ago

NLS (SV40): aa 2-9
3X Flag: aa 15-36
hTtAgo: aa 65-749
NLS (Neoplasmin): aa 775-790
P2A cleavage peptide: aa 797-815

MPKKKRKVGGSGGSDYKDHDGDYKDHDIDYKDDDDKGGGSGGGSGTGGSGGSGGSGGSGGSGRPMNHLGKTEVFLNRF
ALRPLNPEELRPWRLEVVLDPPPGREEVYPLLAQVARRAGGVTVRMGDGLASWSPPEVLVLEGTLARMGQTYAYRLYP
KGRRPLDPKDPGERSVLSALARRLLQERLRRLEGVWVEGLAVYRREHARGPGWRVLGGAVLDLWVSDSGAFLLEVDPA
YRILCEMSLEAWLAQGHPLPKRVRNAYDRRTWELLRLGEEDPKELPLPGGLSLLDYHASKGRLQGREGGRVAWVADPK
DPRKPIPHLTGLLVPVLTLEDLHEEEGSLALSLPWEERRRRTREIASWIGRRLGLGTPEAVRAQAYRLSIPKLMGRRA
VSKPADALRVGFYRAQETALALLRLDGAQGWPEFLRRALLRAFGASGASLRLHTLHAHPSQGLAFREALRKAKEEGVQ
AVLVLTPPMAWEDRNRLKALLLREGLPSQILNVPLREEERHRWENALLGLLAKAGLQVVALSGAYPAELAVGFDAGGR
ESFRFGGAACAVGGDGGHLLWTLPEAQAGERIPQEVVWDLLEETLWAFRRKAGRLPSRVLLLRDGRVPQDEFALALEA
LAREGIAYDLVSVRKSGGGRVYPVQGRLADGLYVPLEDKTFLLLTVHRDFRGTPRPLKLVHEAGDTPLEALAHQIFHL
TRLYPASGFAFPRLPAPLHLADRLVKEVGRLGIRHLKEVDREKLFFVGGSGGSGGSGGSGGSASGGGSGGGSKRPAAT
KKAGQAKKKKGGSGSGATNFSLLKQAGDVEENPGPAAA

S1 Fig. Protein sequences of h*Ng*Ago and h*Tt*Ago used in this study.
