# Supplementary material for: Unexpected binding behaviors of bacterial Argonautes in human cells cast doubts on their use as targetable gene regulators
Source: PLoS One. 2018 Mar 27;13(3):e0193818. doi: 10.1371/journal.pone.0193818 (PMC5870970; doi:10.1371/journal.pone.0193818)
Supplement: S2 Fig — A. hTtAgo was expressed in HEK293 cells and purified by immunoprecipitation (IP) with Flag antibody. A control IP was performed using rabbit IgG. Protein extract from transfected cells was loaded as a control. B. Copurification of nucleic acids with hTtAgo in HEK293 cells. Nucleic acids were resolved on a 15% TBE-urea gel. Nucleic acids were not treated (lane 1) or treated with RNaseA (lane 2) or DNaseI (lane 3). C. 21-nucleotide (nt) DNA guides are complimentary to 98-nt single stranded DNA target. Predicted cleavage sites are indicated by a black triangle. D-F. Purified hTtAgo or hNgAgo were incubated with 21-nt or 24-nt guides, respectively to cleave a 98-nt ssDNA target and run on a 15% TBE-urea gel. Cleavage assays were carried out at 75°C (A), 55°C (B) and 37°C (C). Control Flag-IPs were performed in untransfected HEK293 cells. hTtAgo was incubated with the a DNA guide complimentary to a G nucleotide in position 1 on the target strand (t1G) as indicated. (PDF) [file pone.0193818.s002.pdf]

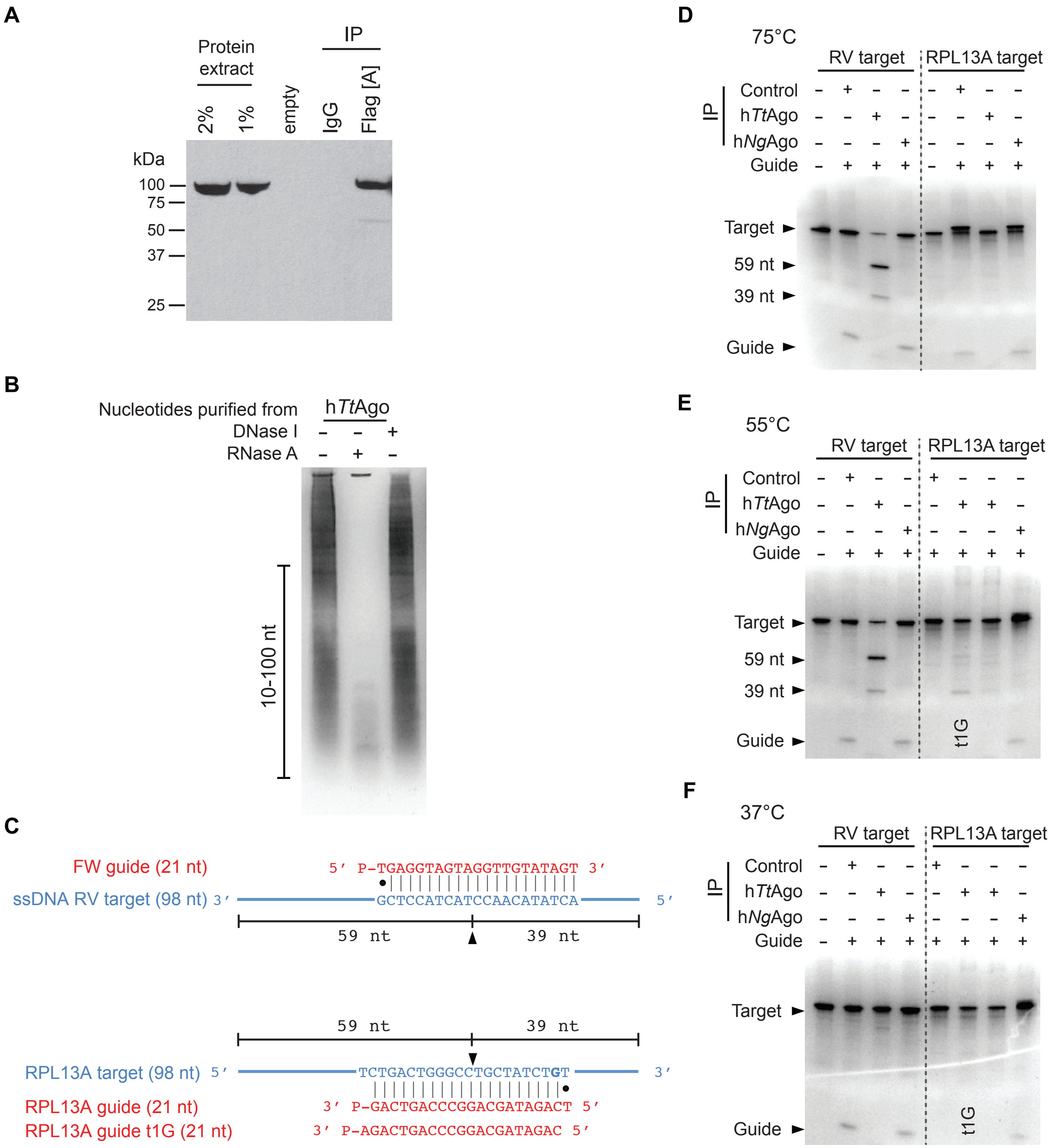

S2 Fig. hTtAgo cleaves ssDNA using ssDNA guides. A. h*Tt*Ago was expressed in HEK293 cells and purified by immunoprecipitation (IP) with Flag antibody. A control IP was performed using rabbit IgG. Protein extract from transfected cells was loaded as a control. **B.** Copurification of nucleic acids with h*Tt*Ago in HEK293 cells. Nucleic acids were resolved on a 15% TBE-urea gel. Nucleic acids were not treated (lane 1) or treated with RNaseA (lane 2) or DNaseI (lane 3). **C**. 21-nucleotide (nt) DNA guides are complimentary to 98-nt single stranded DNA target. Predicted cleavage sites are indicated by a black triangle. **D-F.** Purified h*Tt*Ago or h*Ng*Ago were incubated with 21-nt or 24-nt guides, respectively to cleave a 98-nt ssDNA target and run on a 15% TBE-urea gel. Cleavage assays were carried out at 75°C (**A**), 55°C (**B**) and 37°C (**C**). Control Flag-IPs were performed in untransfected HEK293 cells. h*Tt*Ago was incubated with the a DNA guide complimentary to a G nucleotide in position 1 on the target strand (t1G) as indicated.
