# Supplementary material for: Unexpected binding behaviors of bacterial Argonautes in human cells cast doubts on their use as targetable gene regulators
Source: PLoS One. 2018 Mar 27;13(3):e0193818. doi: 10.1371/journal.pone.0193818 (PMC5870970; doi:10.1371/journal.pone.0193818)
Supplement: S3 Fig — Diagram illustrating the three different gDNA constellations that were tested: forward (FW) guide only, forward (FW) and reverse (RV) guides or forward (FW) and reverse (RV) guides separated by a 15-nt spacer sequence. The genomic RPL13A target site is indicated in blue and complementary ssDNA guides are indicated in red. (PDF) [file pone.0193818.s003.pdf]

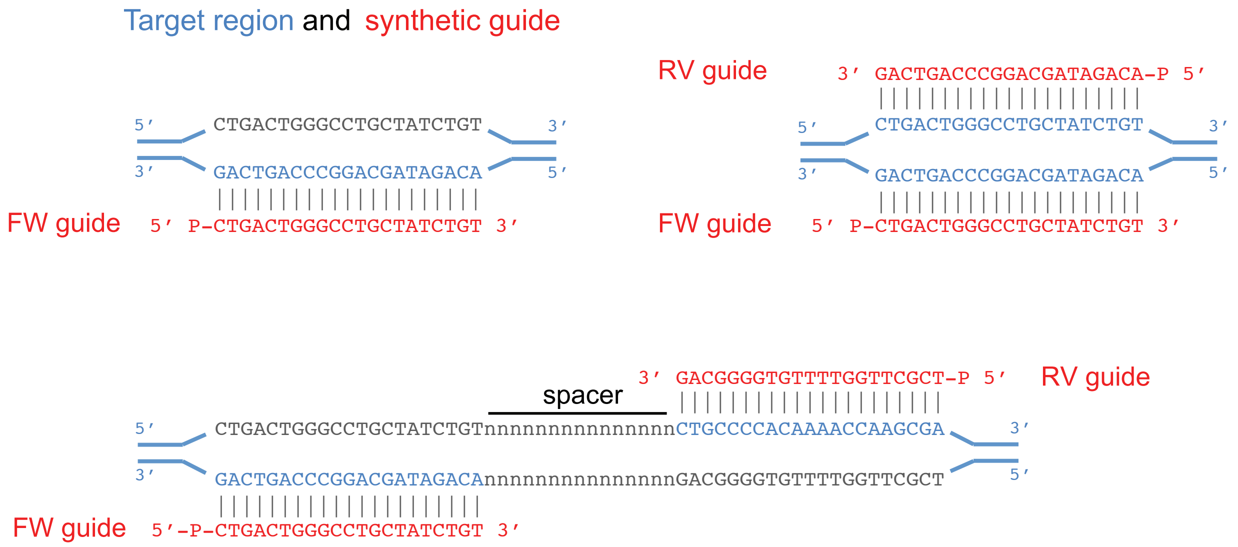

S3 Fig. Design of h*Tt*Ago guide DNAs. Diagram illustrating the three different gDNA constellations that were tested: forward (FW) guide only, forward (FW) and reverse (RV) guides or forward (FW) and reverse (RV) guides separated by a 15-nt spacer sequence. The genomic *RPL13A* target site is indicated in blue and complementary ssDNA guides are indicated in red.
