# Supplementary material for: Unexpected binding behaviors of bacterial Argonautes in human cells cast doubts on their use as targetable gene regulators
Source: PLoS One. 2018 Mar 27;13(3):e0193818. doi: 10.1371/journal.pone.0193818 (PMC5870970; doi:10.1371/journal.pone.0193818)
Supplement: S1 Table — (PDF) [file pone.0193818.s004.pdf]

Supplementary Table S1:
List of oligonucleotide sequences for activity assay

21-nt and 24-nt ss DNA guides were used for h*Tt*Ago and h*Ng*Ago targeting, respectively
Target sites for 21-nt ss DNA guides are underlined

FW guide and RV target according to Swarts et al. 2014

Oligo name	Forward (5'-3')
FW guide (21-nt)	/5Phos/TGAGGTAGTAGGTTGTATAGT
FW guide (24-nt)	/5Phos/GAGGTAGTAGGTTGTATAGTATAT
RV target (98-nt)	TCGACTTTATATTTAAATAATTTAATAT<u>ACTATACAACCTACTACCTC</u>GTATAAATTTTTAAATAAATATTGCATTCAAGCTTTTAATTTAATTAAAT

RPL13A ss DNA guide and ss DNA target

Oligo name	Forward (5'-3')
RPL13A (21-nt)	/5Phos/TCAGATAGCAGGCCCAGTCAG
RPL13A (t1G) (21-nt)	/5Phos/CAGATAGCAGGCCCAGTCAGA
RPL13A (24-nt)	/5Phos/CAGATAGCAGGCCCAGTCAGAGGG
RPL13A target (98-nt)	CCCATGGGCCCACCTCAGTGGGGTGGGTGGGCATCCTTATGAGGCCC<u>TCTGACTGGGCCTGCTATCTG</u>TCACCCAACAGGTATGCTGCCCCACAAAAC
