# Supplementary material for: Unexpected binding behaviors of bacterial Argonautes in human cells cast doubts on their use as targetable gene regulators
Source: PLoS One. 2018 Mar 27;13(3):e0193818. doi: 10.1371/journal.pone.0193818 (PMC5870970; doi:10.1371/journal.pone.0193818)
Supplement: S2 Table — (PDF) [file pone.0193818.s005.pdf]

Supplementary Table S2:
List of oligonucleotide sequences of guide DNAs and gRNA target sites

Oligonucleotide sequences of 21-nt guide DNAs (hTtAgo)
gDNAs are 5'-phosphorylated (5'-p)

	Forward (FW) guides (5'-3')
RPL13A site1	5'-p-CAGAACGCTTCGACCAATGAA
RPL13A site2	5'-p-CTGACTGGGCCTGCTATCTGT
RAB1A site1	5'-p-ACTTTCCAACCCCTGAGTCGC
RAB1A site2	5'-p-CGAGCTGAGGACAGACAGTGC
NPAS1	5'-p-CAAGGCCTCAGGGTACAAGGT
NFE2L1	5'-p-CAGACCCAGTTCCACAACCTG

	Reverse (RV) guides (5'-3')
RPL13A site1	5'-p-TTCATTGGTCGAAGCGTTCTG
RPL13A site2	5'-p-ACAGATAGCAGGCCCAGTCAG
RAB1A site1	5'-p-GCGACTCAGGGGTTGGAAAGT
RAB1A site2	5'-p-GCACTGTCTGTCCTCAGCTCG
NPAS1	5'-p-ACCTTGTACCCTGAGGCCTTG
NFE2L1	5'-p-CAGGTTGTGGAACTGGGTCTG

	15-nt spaced Reverse (15-nt RV) guides (5'-3')
RPL13A site1	5'-p-TGACGCAACGCGGTTGCGCGG
RPL13A site2	5'-p-TCGCTTGGTTTTGTGGGGCAG
RAB1A site1	5'-p-TTTGCAGTGATTATTTATTCA
RAB1A site2	5'-p-GGCGCTTGGCGGGAAGCTGAG
NPAS1	5'-p-GGCTCCTGTATTCCAGGCAGC
NFE2L1	5'-p-CTTGGGGTGGATACCATAGCC

Oligonucleotide sequences of 21-nt (hTtAgo) and 24-nt (hNgAgo) guide DNAs
gDNAs are 5'-phosphorylated

RPL13A site2 (hTtAgo)	5'-p-TCAGATAGCAGGCCCAGTCAG
RPL13A site2 (hNgAgo)	5'-p-GTGACAGATAGCAGGCCCAGTCAG
HER2 (hTtAgo)	5'-p-TTTGTTGGAATGCAGTTGGAG
HER2 (hNgAgo)	5'-p-AGACTTGTTGGAATGCAGTTGGAG
G5 (hNgAgo)	5'-p-CCTACCAGAATCGCCCAGTGGCTG

Oligonucleotide sequences of gRNA target sites (SpCas9)
PAM sequence (5'-NGG-3') is underlined

RPL13A 2-1	CCTTATGAGGCCCTCTGACTGG
RPL13A 2-2	AGATAGCAGGCCCAGTCAGAGG
HER2	GTTGGAATGCAGTTGGAGGGGG
