# Supplementary material for: Unexpected binding behaviors of bacterial Argonautes in human cells cast doubts on their use as targetable gene regulators
Source: PLoS One. 2018 Mar 27;13(3):e0193818. doi: 10.1371/journal.pone.0193818 (PMC5870970; doi:10.1371/journal.pone.0193818)
Supplement: S3 Table — (PDF) [file pone.0193818.s006.pdf]

Supplementary Table S3:
List of oligonucleotide sequences for amplification of genomic target regions

Oligonucleotide sequences for indel analysis

Oligo name	Forward (5'-3')
RPL13A site1	GAGATGAACAGCCCCTCAAA
RPL13A site2	ATGAGATGACTCCACATGCACT
RAB1A site1	CCAAGATACCACTGGGGATG
RAB1A site2	CGAACCAGGAAATAGGGAAG
NPAS1	AGAGGCCAGCCTCACCAA
NFE2L1	TCCAGTTCACCATTCTGCTG

Oligo name	Reverse (5'-3')
RPL13A site1	TGTCGCAGGGTTTCTTATCC
RPL13A site2	TGTAGGTAGTTGCGGAACATCA
RAB1A site1	AGCAATGCAGTGTGCTTTTT
RAB1A site2	AAGTCCTAGCCAGCGAGTCA
NPAS1	GGAGGACTGGACACTGTGTGA
NFE2L1	CCAGGCATTTACCTCAGTGG

Oligonucleotide sequences for indel analysis

Barcodes at 5' end of Forward primer are underlined

Oligo name	Forward (5'-3')
HER2 Ampl-1 SEQ F	ATCACGCGAAGAGAGGGAGAAAGTG
HER2 Ampl-2 SEQ F	CGATGGCGAAGAGAGGGAGAAAGTG
RPL13A Ampl-3 SEQ F	TTAGGTGACTCCACATGCACTACCA
RPL13A Ampl-4 SEQ F	TGACCTGACTCCACATGCACTACCA

Oligo name	Reverse (5'-3')
HER2 Ampl SEQ R	GGGCTCCCCTGGTTTCTC
RPL13A Ampl SEQ R	GGCATAGCTCACCTTGTCGT

Oligonucleotide sequences for ChIP-qPCR and ChIP-PCR

Oligo name	Forward (5'-3')
RPL13A PCR-F	GGTGGGTGGGCATCCTTAT
GAPDH PCR-F	CACCGTCAAGGCTGAGAACG
G5 PCR-F	GCAATCAGGCCTACCAGAAT
HER2 PCR-F	TTGGAATGCAGTTGGAGGGG
EPCAM PCR-F	CCCAACTCCCGGGCGGTGAC

Oligo name	Reverse (5'-3')
RPL13A PCR-R	CTGGCCTCGCTTGGTTTT
GAPDH PCR-R	ATACCCAAGGGAGCCACACC
G5 PCR-R	GGGATTGGAGTAGACGGTCA
HER2 PCR-R	GGTTTCTCCGGTCCCAATGG
EPCAM PCR-R	GGGTCCGCGTCGGGAGGACA
