# Supplementary material for: Unexpected binding behaviors of bacterial Argonautes in human cells cast doubts on their use as targetable gene regulators
Source: PLoS One. 2018 Mar 27;13(3):e0193818. doi: 10.1371/journal.pone.0193818 (PMC5870970; doi:10.1371/journal.pone.0193818)
Supplement: S4 Table — (PDF) [file pone.0193818.s007.pdf]

Supplementary Table S4:
Indel analysis of PCR amplified genomic target sites using 5'-phosphorylated guide DNAs

gDNA: /5Phos/NNNNNNNNNNNNNNNNNNNNN

% Indels = (Indels+Deletions)/Reads*100

NFE2L1

Cell type	Treatment	% Indel	# Reads	Insertions	Deletions	Substitutions	% Substitutions
293	Untreated	0.00	9,756	0	0	47	0.48
	hTtAgo	0.00	28,628	0	0	154	0.54
	hTtAgo / FW gDNA	0.00	28,955	0	0	123	0.42
	hTtAgo / FW+RV gDNA	0.00	13,293	0	0	80	0.60
	hTtAgo / FW+ 15-nt RV gDNA	0.00	20,755	0	0	91	0.44
Hela	Untreated	0.01	14,331	1	1	76	0.53
	hTtAgo	0.00	18,014	0	0	92	0.51
	hTtAgo / FW gDNA	0.00	10,173	0	0	53	0.52
	hTtAgo / FW+RV gDNA	0.01	8,947	0	1	43	0.48
	hTtAgo / FW+ 15-nt RV gDNA	0.00	6,841	0	0	44	0.64

NPAS1

Cell type	Treatment	% Indel	# Reads	Insertions	Deletions	Substitutions	% Substitutions
293	Untreated	0.01	12,605	0	1	82	0.65
	hTtAgo	0.00	31,862	0	1	213	0.67
	hTtAgo / FW gDNA	0.00	44,133	0	1	319	0.72
	hTtAgo / FW+RV gDNA	0.01	15,995	0	1	104	0.65
	hTtAgo / FW+ 15-nt RV gDNA	0.00	53,417	0	1	392	0.73
Hela	Untreated	0.01	15,641	0	1	110	0.70
	hTtAgo	0.00	23,258	0	0	189	0.81
	hTtAgo / FW gDNA	0.00	28,163	0	0	195	0.69
	hTtAgo / FW+RV gDNA	0.01	18,405	0	1	130	0.71
	hTtAgo / FW+ 15-nt RV gDNA	0.00	20,408	0	0	120	0.59

RAB1A site1

Cell type	Treatment	% Indel	# Reads	Insertions	Deletions	Substitutions	% Substitutions
293	Untreated	0.01	15,716	1	0	173	1.10
	hTtAgo	0.00	34,405	0	1	287	0.83
	hTtAgo / FW gDNA	0.00	46,837	0	2	422	0.90
	hTtAgo / FW+RV gDNA	0.00	26,199	0	1	258	0.98
	hTtAgo / FW+ 15-nt RV gDNA	0.01	49,392	0	5	525	1.06
Hela	Untreated	0.01	25,129	0	2	253	1.01
	hTtAgo	0.01	30,950	0	2	248	0.80
	hTtAgo / FW gDNA	0.00	29,750	0	0	249	0.84
	hTtAgo / FW+RV gDNA	0.00	20,158	0	1	212	1.05
	hTtAgo / FW+ 15-nt RV gDNA	0.00	19,748	0	0	193	0.98

RAB1A site2

Cell type	Treatment	% Indel	# Reads	Insertions	Deletions	Substitutions	% Substitutions
293	Untreated	0.000	20,581	0	0	149	0.72
	hTtAgo	0.000	26,958	0	0	162	0.60
	hTtAgo / FW gDNA	0.000	35,014	0	0	208	0.59
	hTtAgo / FW+RV gDNA	0.000	26,282	0	0	162	0.62
	hTtAgo / FW+ 15-nt RV gDNA	0.000	43,979	0	0	285	0.65
Hela	Untreated	0.000	40,071	0	0	234	0.58
	hTtAgo	0.000	37,720	0	0	223	0.59
	hTtAgo / FW gDNA	0.000	49,148	0	0	303	0.62
	hTtAgo / FW+RV gDNA	0.000	39,481	0	0	242	0.61
	hTtAgo / FW+ 15-nt RV gDNA	0.000	49,554	0	0	369	0.74

RPL13A site1

Cell type	Treatment	% Indel	# Reads	Insertions	Deletions	Substitutions	% Substitutions
293	Untreated	0.010	10,003	0	1	43	0.43
	hTtAgo	0.000	22,616	0	0	117	0.52
	hTtAgo / FW gDNA	0.004	22,324	0	1	90	0.40
	hTtAgo / FW+RV gDNA	0.000	10,905	0	0	41	0.38
	hTtAgo / FW+ 15-nt RV gDNA	0.000	28,859	0	0	128	0.44
Hela	Untreated	0.006	15,987	0	1	71	0.44
	hTtAgo	0.000	15,324	0	0	70	0.46
	hTtAgo / FW gDNA	0.006	16,840	0	1	77	0.46
	hTtAgo / FW+RV gDNA	0.006	17,919	0	1	73	0.41
	hTtAgo / FW+ 15-nt RV gDNA	0.000	13,073	0	0	46	0.35

RPL13A site2

Cell type	Treatment	% Indel	# Reads	Insertions	Deletions	Substitutions	% Substitutions
293	Untreated	0.000	6,432	0	0	28	0.44
	hTtAgo	0.000	15,418	0	0	55	0.36
	hTtAgo / FW gDNA	0.000	23,771	0	0	111	0.47
	hTtAgo / FW+RV gDNA	0.000	12,123	0	0	53	0.44
	hTtAgo / FW+ 15-nt RV gDNA	0.000	23,440	0	0	103	0.44
Hela	Untreated	0.000	15,154	0	0	75	0.49
	hTtAgo	0.000	15,278	0	0	66	0.43
	hTtAgo / FW gDNA	0.007	14,771	1	0	61	0.41
	hTtAgo / FW+RV gDNA	0.000	15,136	0	0	70	0.46
	hTtAgo / FW+ 15-nt RV gDNA	0.000	12,009	0	0	60	0.50
