# Supplementary material for: Unexpected binding behaviors of bacterial Argonautes in human cells cast doubts on their use as targetable gene regulators
Source: PLoS One. 2018 Mar 27;13(3):e0193818. doi: 10.1371/journal.pone.0193818 (PMC5870970; doi:10.1371/journal.pone.0193818)
Supplement: S5 Table — (PDF) [file pone.0193818.s008.pdf]

Supplementary Table S5:
Indel analysis of amplified genomic targets using 5'-phosphorylated gDNAs with phosphorothioate modifications

gDNA: /5Phos/N*N*NNNNNNNNNNNNNNNNN*N*N
*** phosphorothioate linkage**

% Indels = (Indels+Deletions)/Reads*100

NFE2L1

Cell type	Treatment	% Indel	# Reads	Insertions	Deletions	Substitutions	% Substitutions
293	Untreated	0.00	9,756	0	0	47	0.48
	hTtAgo	0.00	28,628	0	0	154	0.54
	hTtAgo / FW gDNA	0.00	13,851	0	0	54	0.39
	hTtAgo / FW+RV gDNA	0.00	11,732	0	0	53	0.45
	hTtAgo / FW+ 15-nt RV gDNA	0.00	10,813	0	0	71	0.66

NPAS1

Cell type	Treatment	% Indel	# Reads	Insertions	Deletions	Substitutions	% Substitutions
293	Untreated	0.01	12,605	0	1	82	0.65
	hTtAgo	0.00	31,862	0	1	213	0.67
	hTtAgo / FW gDNA	0.00	18,432	0	0	119	0.65
	hTtAgo / FW+RV gDNA	0.01	18,890	0	1	118	0.62
	hTtAgo / FW+ 15-nt RV gDNA	0.01	18,432	0	1	126	0.68

RAB1A site1

Cell type	Treatment	% Indel	# Reads	Insertions	Deletions	Substitutions	% Substitutions
293	Untreated	0.01	15,716	1	0	173	1.10
	hTtAgo	0.00	34,405	0	1	287	0.83
	hTtAgo / FW gDNA	0.01	27,472	0	3	254	0.92
	hTtAgo / FW+RV gDNA	0.00	20,193	0	0	162	0.80
	hTtAgo / FW+ 15-nt RV gDNA	0.00	22,219	0	1	191	0.86

RAB1A site2

Cell type	Treatment	% Indel	# Reads	Insertions	Deletions	Substitutions	% Substitutions
293	Untreated	0.00	20,581	0	0	149	0.72
	hTtAgo	0.00	26,958	0	0	162	0.60
	hTtAgo / FW gDNA	0.00	16,533	0	0	104	0.63
	hTtAgo / FW+RV gDNA	0.00	15,114	0	0	92	0.61
	hTtAgo / FW+ 15-nt RV gDNA	0.00	20,802	0	1	135	0.65
Hela	Untreated	0.00	40,071	0	0	234	0.58
	hTtAgo	0.00	37,720	0	0	223	0.59
	hTtAgo / FW gDNA	0.00	27,775	0	0	155	0.56
	hTtAgo / FW+RV gDNA	0.00	23,040	0	0	141	0.61
	hTtAgo / FW+ 15-nt RV gDNA	0.00	37,893	0	1	181	0.48

RPL13A site1

Cell type	Treatment	% Indel	# Reads	Insertions	Deletions	Substitutions	% Substitutions
293	Untreated	0.01	10,003	0	1	43	0.43
	hTtAgo	0.00	22,616	0	0	117	0.52
	hTtAgo / FW gDNA	0.01	9,753	0	1	47	0.48
	hTtAgo / FW+RV gDNA	0.01	9,011	0	1	38	0.42
	hTtAgo / FW+ 15-nt RV gDNA	0.00	7,130	0	0	39	0.55
Hela	Untreated	0.01	15,987	0	1	71	0.44
	hTtAgo	0.00	15,324	0	0	70	0.46
	hTtAgo / FW gDNA	0.03	10,805	0	3	55	0.51
	hTtAgo / FW+RV gDNA	0.03	8,703	0	3	31	0.36
	hTtAgo / FW+ 15-nt RV gDNA	0.01	13,238	0	1	61	0.46

RPL13A site2

Cell type	Treatment	% Indel	# Reads	Insertions	Deletions	Substitutions	% Substitutions
293	Untreated	0.00	6,432	0	0	28	0.44
	hTtAgo	0.00	15,418	0	0	55	0.36
	hTtAgo / FW gDNA	0.00	10,567	0	0	44	0.42
	hTtAgo / FW+RV gDNA	0.00	7,973	0	0	31	0.39
	hTtAgo / FW+ 15-nt RV gDNA	0.00	9,244	0	0	36	0.39
Hela	Untreated	0.00	15,154	0	0	75	0.49
	hTtAgo	0.00	15,278	0	0	66	0.43
	hTtAgo / FW gDNA	0.00	13,756	0	0	52	0.38
	hTtAgo / FW+RV gDNA	0.00	13,253	0	0	52	0.39
	hTtAgo / FW+ 15-nt RV gDNA	0.00	17,830	0	0	74	0.42
