# Supplementary material for: Unexpected binding behaviors of bacterial Argonautes in human cells cast doubts on their use as targetable gene regulators
Source: PLoS One. 2018 Mar 27;13(3):e0193818. doi: 10.1371/journal.pone.0193818 (PMC5870970; doi:10.1371/journal.pone.0193818)
Supplement: S6 Table — (PDF) [file pone.0193818.s009.pdf]

Supplementary Table S6:
Indel analysis of amplified genomic target sites comparing cleavage ability of RNA-guided SpCas9 and DNA-guided TtAgo and NgAgo in HeK293-c18 cells.

% Indels = (Indels+Deletions)/Reads*100

Target locus	Expressed protein	gDNA/gRNA	% Indel	# Reads	Insertions	Deletions	Substitutions	% Substitutions
Her2	mCherry	-	0.07	15,248	3	7	73	0.48
	Cas9	empty	0.05	6,139	1	2	31	0.50
	Cas9	gRNA	39.88	7,525	578	2423	118	1.57
	TtAgo	-	0.08	10,533	4	4	18	0.17
	TtAgo	gDNA	0.00	12,059	0	0	19	0.16
	TtAgo	2XgDNA	0.01	7,637	0	1	13	0.17
	NgAgo	-	0.01	9,226	0	1	14	0.15
	NgAgo	gDNA	0.01	20,421	2	1	31	0.15
	NgAgo	2XgDNA	0.03	15,440	1	3	19	0.12
RPL13A	mCherry	-	0.00	6,264	0	0	17	0.27
	Cas9	empty	0.00	7,145	0	0	23	0.32
	Cas9	gRNA2-1	25.01	6,585	1464	183	32	0.49
	Cas9	gRNA2-2	43.17	5,978	2292	289	35	0.59
	TtAgo	-	0.00	8,177	0	0	29	0.35
	TtAgo	gDNA	0.00	4,556	0	0	8	0.18
	TtAgo	2XgDNA	0.00	7,446	0	0	28	0.38
	NgAgo	-	0.02	6,457	0	1	25	0.39
	NgAgo	gDNA	0.00	8,644	0	0	30	0.35
	NgAgo	2XgDNA	0.00	12,466	0	0	53	0.43
